# *Mycobacterium avium* Subspecies *paratuberculosis* Infects and Replicates within Human Monocyte-Derived Dendritic Cells

**DOI:** 10.3390/microorganisms8070994

**Published:** 2020-07-03

**Authors:** William D. Rees, Ana C. Lorenzo-Leal, Theodore S. Steiner, Horacio Bach

**Affiliations:** 1 Department of Medicine, Division of Infectious Diseases, University of British Columbia, Vancouver, BC V5Z3J5, Canada; wdrees@alumni.ubc.ca (W.D.R.); ana.lorenzoll@udlap.mx (A.C.L.-L.); 2 BC Children’s Hospital Research Institute, Vancouver, BC V6H3N1, Canada; 3 Chemical and Food Engineering Department, Universidad de las Americas Puebla, San Andres Cholula, Puebla 72810, Mexico

**Keywords:** *Mycobacterium avium* subspecies *paratuberculosis*, dendritic cells, protein tyrosine phosphatase, Crohn’s disease, Johne’s disease

## Abstract

Background: *Mycobacterium avium* subspecies *paratuberculosis* (MAP), a member of the mycobacteriaceae family, causes Johne’s disease in ruminants, which resembles Crohn’s disease (CD) in humans. MAP was proposed to be one of the causes of human CD, but the evidence remains elusive. Macrophages were reported to be the only cell where MAP proliferates in ruminants and humans and is likely the major producer of TNFα-associated inflammation. However, whether human dendritic cells (DCs), another major antigen-presenting cell (APC), have the ability to harbor MAP and disseminate infection, remains unknown. Methods: Human monocyte-derived dendritic cells (moDCs) were infected with MAP and phagocytosis and intracellular survival were quantified by immunofluorescence (IF) and colony counts, respectively. MoDC cytokine expression was measured via ELISA and their activation state was measured via flow cytometry. Results: We showed that MAP can infect and replicate in human moDCs as means to evade the immune system for successful infection, through inhibition of the phago-lysosome fusion via the secretion of protein tyrosine phosphatase PtpA. This mechanism initially led to a state of tolerance in moDCs and then subsequently caused a pro-inflammatory response as infection persisted, characterized by the upregulation of IL-6 and TNFα, and downregulation of IL-10. Moreover, we showed that moDCs have the ability to phagocytose up to 18% of MAP, when exposed at a multiplicity of infection of 1:1. Conclusion: Infection and subsequent proliferation of MAP within moDCs could provide a unique means for the dissemination of MAP to lymphoid tissue, while altering immune responses to facilitate the persistence of infection of host tissues in CD.

## 1. Introduction

*Mycobacterium avium* subspecies *paratuberculosis* (MAP) is a member of the mycobacteriaceae family, and is related to *Mycobacterium tuberculosis* (Mtb) and *Mycobacterium leprae*, both human pathogenic organisms. MAP causes Johne’s disease in ruminants, which primarily involves the small intestine, causing diarrhea, wasting, weight loss, bloody stool, and eventually death [1]. As this pathology resembles Crohn’s disease (CD) in humans, MAP was proposed to be one of the causes of human CD [2] but definitive evidence remains elusive, due to the difficulties in isolating and culturing live MAP from biopsies in CD patients. However, there is some evidence linking MAP infection to the development of CD. For example, biopsies from both the small intestine (SI) and colon of CD and irritable bowel syndrome (IBS) patients was shown to have detectable MAP DNA levels [3,4,5] and MAP was cultured via isolation from the blood, milk [6], or serum of patients with CD [7,8,9]. TNFα, a cytokine found to be a driving factor in CD (and a target of successful mAb therapies), was also studied in the context of MAP infection, and was shown to be elevated in MAP-positive CD patients, [10] likely due to the secretion of TNFα via macrophage [11,12]. Moreover, two single nucleotide polymorphisms (SNPs) known to affect clinical outcomes in CD patients, *TNFRSF1A:rs767455* and *TNFRSF1B:rs3397,* were associated with high susceptibility to MAP infection [13].

Macrophages were reported as the only cells where MAP proliferates in ruminants and humans [14,15]. Since macrophage and dendritic cells (DCs) are the main antigen-presenting cells (APCs) that initiate an adaptive immune response, and are important in the production of TNFα, several studies examined DC maturation upon MAP stimulation. A few studies demonstrated that the MAP proteins MAP1981c [16] and malate dehydrogenase (MDH) [17], mature DCs to produce a Th1 immunity environment, via upregulation of IL-6, TNFα, IFNγ, and IL-1ß. Another MAP protein, the nicotinate-nucleotide-dimethylbenzimidazole phosphoribosyl transferase CobT, was shown to cause activation of the toll-like receptor 4 (TLR4), resulting in Th1 cell expansion [18]. Others demonstrated DC maturation impairment when DCs were incubated in supernatants from MAP-infected macrophage [19].

MAP was shown to infect but not proliferate within the human enterocyte cell line Caco-2 [20] and invade ileal Peyer’s Patches (IPP) to evade mucosal immune responses [21]. This suggests that MAP is able to evade the host immune system and further drive disease.

MAP was shown to exclusively infect and replicate inside of macrophage, and to have evolved to evade macrophage defense mechanisms by different means, such as altering phagosome acidification for its survival [22], phagosome-lysosome fusion inhibition via secretion of mycobacterial tyrosine phosphatase PtpA [23], and phagosome-lysosome arrest via protein kinase G (PknG), as demonstrated in *Mycobacterium tuberculosis* (Mtb) [24]. These data demonstrate that MAP utilizes macrophage to proliferate and alter host immune responses. However, macrophage are mostly resident immune cells, whereas DCs are migratory. DC infection and subsequent proliferation of MAP could provoke a more severe systemic immune response, which is why it is of importance to understand the role of DC infection in MAP-related diseases. This is the first study, to our knowledge, to provide evidence that MAP infects and replicates within human monocyte-derived DCs (moDCs), initially leading to a state of tolerance in DCs upon infection/replication, and then subsequently leading to a pro-inflammatory response as infection persists, as measured by the upregulation of pro-inflammatory cytokines, as well as the T-cell priming receptors.

## 2. Materials and Methods

### 2.1. Cell and Culture

Human blood was obtained from donors through the NETCad Blood-for-Research program (Canadian Blood Services), under an ethics protocol approved through the University of British Columbia Clinical Research Ethics Board (H16-00927; approved on June 23, 2016).

The strain MAP k-10 (ATCC BAA-962, Manassas, VA, USA) was used in this study. Bacteria were cultured in 7H9 broth (Becton & Dickinson, Franklin Lakes, NJ, USA) supplemented with 0.05% Tween-80 (Fisher Scientific, Waltham, MA, USA), 10% OADC, and 2 mg/L mycobactin J (Allied Monitor, IN, USA). OADC was prepared by mixing 25 mg of oleic acid (Sigma-Aldrich, St. Louis, MO, USA), 2.5 g bovine serum albumin (BSA, VWR, Radnor, PA, USA), 1 g dextrose (VWR), 2 mg of catalase (Sigma-Aldrich), and 425 mg of NaCl (Fisher), in a final volume of 50 mL. Bacteria were used when the OD at 600 nm was around 0.7.

MoDCs were generated from monocytes (collected from donors) enriched from buffy coats via Ficoll (Sigma-Aldrich) gradient and plate adherence, for 1–2 h, as described [25]. MoDCs were fed twice (day 1 and day 4) with RPMI media containing 10% human serum (Thermo Fisher Scientific, Waltham, MA, USA), 50 ng/mL IL-4 (StemCell, Vancouver, Canada), and 100 ng/mL granulocyte-macrophage colony-stimulating factor (GM-CSF, StemCell). Cells (18,000 cells/treatment) were harvested, counted, and cultured/stimulated with either live MAP, antibiotic-killed MAP (KMAP) using 100 μg/mL kanamycin (Fisher) overnight at 37 °C [26], LPS (1 μg/mL, Sigma-Aldrich), or LPS+MAP, and the supernatants were collected for ELISA or flow cytometry analysis, at time points 2, 24, and 48 h. To validate the killing of MAP, cells were plated on solidified 7H9 (B&D), supplemented with 10% OADC (B&D) and mycobactin J (Allied Monitor) and placed at 37 °C. MAP was considered killed when no colonies were observed after 2 months of incubation. Prior to the stimulation, live MAP were washed with PBS (×3) and opsonized with 10% human AB^+^ serum (Thermo Fisher) in RPMI for 30 min, at 37 °C. MoDCs were infected at a MOI of 1:1 and non-internalized bacteria were killed by adding 50 μg/mL amikacin (Sigma-Aldrich) after 1 h post-infection. The media was replaced with RPMI and human serum only for the next few days.

### 2.2. Phagocytosis Index

Live MAP were stained with 10 μg/mL Rhodamine B (Sigma-Aldrich) for 1 h, washed with PBS, and opsonized, as detailed above [14]. MoDCs (18,000 cells/well) were infected using a multiplicity of infection of 1:1 for 2 h. Cells were collected and dispensed on a microscope slide, covered by a cover slip, and sealed after 24 h. The phagocytosis index was calculated as the number of infected cells/total number of cells, per microscope field × 100. A total of 300 cells were counted for each donor.

### 2.3. Flow Cytometry

For flow cytometry analysis, moDCs were harvested, washed, stained, and analyzed by flow cytometry on a Fortessa cytometer (B&D). Live moDCs were defined by sequential gating by forward/side scatter, BV510 viability dye, single cells (SSC-A/SSC-H), and finally CD11b and CD11c (Figure S1). The CD11c^+^/CD11b^+^ population was analyzed for CD80, CD86, CD103, and MHCII, as described in [27]. The mean fluorescence intensity (MFI) of the aforementioned markers were normalized to the MFI from DCs that were unstimulated. MoDCs matured overnight with LPS (1 µg/mL) and FliC (1 µg/mL) were used as a positive control for staining for surface marker antibodies. The following antibodies were used: CD80 (clone 2D10.4), MHCII/HLA-DR (clone LN3), CD86 (clone IT2.2), CD11c (clone 3.9), and BV506 viability dye (Thermo Fisher), whereas CD103 (clone Ber-ACT8), and CD11b (clone ICRF44) were from B&D. All experiments were performed in triplicate, with three to four different blood donors.

### 2.4. ELISA

For the cytokine ELISAs, kits from B&D were used according to the manufacturer’s instructions. All experiments were performed in triplicate.

### 2.5. MAP Colony Counting

For the colony-forming units (CFUs) counting, samples were washed with PBS (×3) and plated on 7H10 (B&D) plates supplemented with 10% OADC (B&D) and mycobactin J (2 mg/L, Alled Monitor), after performing serial dilutions in PBS. CFUs were counted when the colonies were visible (~45 days post-plating). All experiments were performed in triplicate with three biological replicates obtained from different blood donors.

### 2.6. Immunofluorescence

Immunofluorescence analysis, including the antibodies used in the experiment, was performed as published [14], but using moDCs isolated via the monocyte adherence step, with IL-4 and GM-CSF supplementation, as described earlier.

### 2.7. Statistical Analyses

Statistical comparisons were run using the GraphPad Prism Software v.7 (San Diego, CA, USA). Multiple comparisons were done using nonparametric analysis (Kruskal-Wallis) or one-way or two-way ANOVA with Tukey’s multiple comparison, as indicated. Single comparisons were done by *t*-test. Data are represented as mean ± SEM, unless otherwise indicated.

## 3. Results

### 3.1. MAP Infects and Replicates Inside moDCs

**Figure 1:**
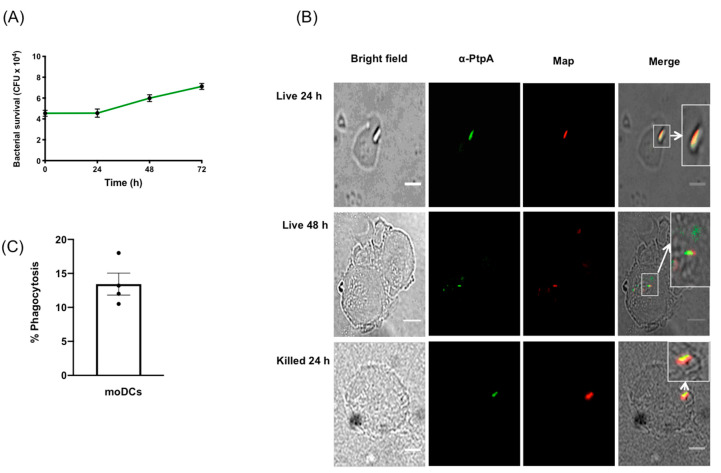
*Mycobacterium avium* subspecies *paratuberculosis* (MAP) infects and replicates in human monocyte derived dendritic cells and expresses PtpA. (**A**) Human moDCs were exposed to MAP and harvested at 24 h intervals for 72 h. Cell lysates were plated on MAP culture media and the colony-forming units (CFUs) were counted, after 8 weeks of growth. Error bars are the mean ± SEM of *n* = 3 DC blood donors analyzed in triplicate. (**B**) moDCs were processed and imaged for the protein phosphatase PtpA (green) and MAP (red), at 24 and 48 h post-exposure to MAP. Images representative of experiments using *n* = 3 independent DC blood donors. (**C**) Phagocytosis index expressed in percentages of *n* = 300. Scale bar = 20 µm

Due to the difficulty in obtaining adequate numbers of viable DCs from primary human intestinal tissue biopsies, human moDCs were differentiated from monocytes through growth in GM-CSF and IL-4 [25]. Non-adherent cells were exposed to live MAP at an MOI of 1:1, and survival, growth, and phagocytosis were measured by harvesting the moDCs, daily for 72 h, and subsequently plating to count the MAP CFUs. We measured the ability of kanamycin-treated KMAP or live MAP to kill moDCs upon infection, by measuring cell viability via flow cytometry on the CD11c^+^CD11b^+^ population, which was ~96% homogenous in our experiments [27] (Figure S1). We found that MAP, but not KMAP, caused a small but statistically significant decrease in cell viability (85.9% for *T* = 0 and 24 h, and 89.3% for *T* = 48 h), compared to the untreated moDCs (Figure S2). Nonetheless, we found that MAP survived and likely replicated in moDCs over time, starting with 4 × 10^4^ CFU at *T* = 0 and increasing to 7 × 10^4^ CFU at *T* = 72 h post infection (Figure 1A). Moreover, MAP was phagocytosed up to 18% (mean of 13.25%) by moDCs (Figure 1C), suggesting a mechanism through which live MAP could enter moDCs for a successful infection.

The mycobacterial protein phosphatase PtpA was secreted within macrophages upon infection with MAP and Mtb [14,23]. PtpA inhibited the phago-lysosome fusion and phagosome acidification functions in macrophages [23,28]. To determine whether MAP secreted PtpA in infected moDCs, an immunofluorescence co-localization was performed (Figure 1B) in the cells, post-MAP infection, at 24 h and 48 h. Results indicated that PtpA (in green) co-localized at 24 h and 48 h post-infection with MAP (in red), suggesting that MAP used similar PtpA secretion mechanisms in moDCs, as in the MAP and Mtb-infected macrophage.

### 3.2. MAP Infection Delays moDC Maturation

**Figure 2:**
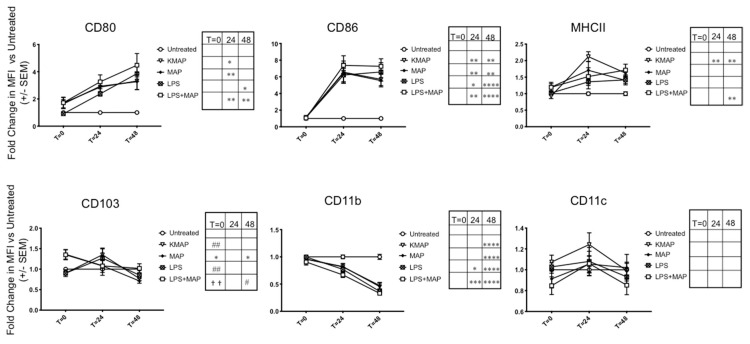
MAP infection delays maturation of moDCs. moDCs (1.8 × 10^4^ cells) were untreated or infected/treated with the same number of opsonized MAP, antibiotic-killed MAP (KMAP), MAP+LPS, or LPS alone for *T* = 0 (2 h), *T* = 24 h, and *T* = 48 h. MoDCs were stained for CD80, CD86, CD11b, CD11c, CD103, and MHCII and the mean fluorescence intensity (MFI) of CD80, CD86, MHCII, CD103, CD11b, and CD11c within the CD11c^+^CD11b^+^ population was calculated, and normalized to the MFI, from cells in the same experiment that were untreated. * significance vs. untreated: * *p* < 0.05; ** *p* < 0.01; *** *p* < 0.001; *p* < 0.0001 ****. # significance vs. MAP: # *p* < 0.05; ## *p* < 0.01. † Significance vs. KMAP: †† *p* < 0.01 (one-way ANOVA with multiple comparisons). Shown is the average ± SEM of *n* = 3–4 DC blood donors analyzed in triplicates.

Since MAP produces the proteins MAP1981c [16] and MDH [17], which were shown to mature DCs, we hypothesized that infection of MAP in moDCs would also mature DCs in a similar fashion. To assess moDC maturation status upon MAP infection, we examined the expression of T-cell priming receptors CD80/86, as well as MHCII for antigen presentation, and CD103, which was expressed on tolerogenic DCs [29], via flow cytometry at 2 h, 24 h, and 48 h post-infection of untreated immature moDCs, gating on the live, CD11c^+^/CD11b^+^ population. A representative experiment of *T* = 24 h MFI shifts are shown in Figure S3. Upon initial infection, we found a small but non-significant increase in CD80 with live MAP or KMAP, which increased significantly at 24 h and 48 h. CD86 expression was also significantly increased 24 h and 48 h after infection with live MAP or KMAP. Upon initial infection, we measured a significant increase in the CD103 expression in live MAP infected moDCs vs. untreated and KMAP conditions (1.36 ± 0.12 and 1.51 ± 0.24 fold change, respectively), as well as untreated vs. MAP+LPS treated moDCs (1.35 ± 0.12 fold increase; Figure 2). In contrast, LPS or KMAP did not significantly increase CD103 expression, compared to untreated moDCs. As infection proceeded, CD103 began to significantly down-regulate only in MAP-infected moDCs. Interestingly, MHCII expression was significantly increased in only moDCs treated with KMAP at 24 h (2.2 ± 0.15-fold increase vs. unstimulated), as well as KMAP and LPS+MAP (1.6 ± 0.10 and 1.7 ± 0.18-fold increase vs. unstimulated, respectively) at 48 h, but not live MAP alone, which was consistent with bovine macrophage infection with MAP [30]. We also measured expression of the DC integrins CD11b and CD11c, and demonstrated that CD11b, but not CD11c, was significantly down-regulated over time with live or killed MAP.

Overall, the similar moDC responses to live MAP, KMAP, and LPS suggest that it is likely that surface pattern recognition receptors (PRRs) on MAP acting through TLR pathways lead to alterations of the moDC function. The only exception was with MHCII, where live MAP caused less of an upregulation in MHCII, consistent with inhibition of phago-lysosome maturation caused by MAP virulence factors. This in turn could reduce immune surveillance by reducing MAP antigen presentation on moDCs.

### 3.3. MAP Infection of moDCs Causes Transient Cytokine Production

**Figure 3:**
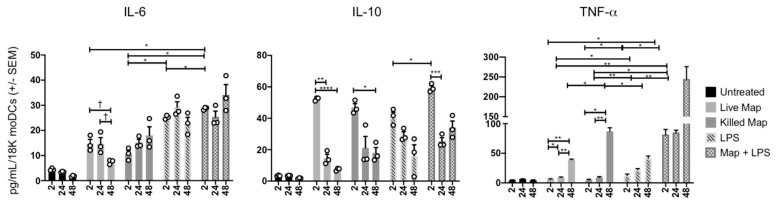
MAP-infected moDCs display a downregulated pro-inflammatory phenotype. MoDCs (1.8 × 10^4^ cells) were untreated or infected/treated with the same number of opsonized MAP, killed MAP (KMAP), or LPS alone for *T* = 0 (2 h), *T* = 24 h, and *T* = 48 h. Supernatants were collected and ELISA for IL-6, IL-10, and TNFα was performed. * *p* < 0.05; ** *p* < 0.01; *** *p* < 0.001; *p* < 0.0001 **** as calculated using two-way ANOVA with multiple comparisons; † *p* < 0.05, *t*-test. Shown is the average ± SEM of *n* = 3–4 DC blood donors analyzed in triplicate.

MoDC cytokine secretion was measured in parallel with replication experiments, to better understand the cytokine milieu during infection (Figure 3). Live and KMAP caused rapid and similar production of IL-6 and IL-10, although less than the positive control, LPS. IL-10 production rapidly decreased over 24–48 h in all conditions, while IL-6 remained stable or increased in all conditions other than the live MAP infection. In contrast, TNFα production was detected rapidly only with combined LPS and MAP stimulation, but was increased at 48 h, in all conditions. Addition of MAP to LPS enhanced early production of all three cytokines. At 48 h, live MAP caused significantly less IL-6 and TNFα production than KMAP, suggesting a potential anti-inflammatory activity of live MAP on moDCs, unless LPS was concomitantly present. These data suggest that MAP infected moDCs displayed an initial tolerogenic state, possibly to hide from the immune system, then subsequently increased their pro-inflammatory potential over time.

## 4. Discussion

MAP is the etiological agent of Johne’s disease in ruminants and there is a growing body of evidence that suggests that MAP might play a role in the development of IBD [2,31] as well as exacerbating dextran-sulfate sodium (DSS) induced colitis in mouse models [32,33]. MAP is an intracellular pathogenic organism that might live for up to a year in the environment, after passing via infected animals [34,35], and can survive in milk [36,37] and its derivatives [38,39] with a potential transmission to humans via contamination of dairy products. Macrophage were reported as the only cells where MAP proliferated in ruminants and humans [14,15], and likely are the key APC driving the production of TNFα in MAP-positive patients [13]. However, since macrophage are mainly tissue resident immune cells, it is important to understand if DCs can be used as a vehicle of MAP replication/dissemination, since DCs are migratory and would lead to a potentially more widespread systemic immune response. The only prior studies to examine MAP in the context of DC immune responses only measured PRR stimulation via MAP proteins [16,17,18] and showed that MAP-stimulated DCs produce a Th1-like environment.

Our study was the first to show that MAP can infect and replicate in human-derived moDCs to evade the immune system for successful infection. We found that MAP survived and proliferated in moDCs over at least 72 h. This was associated with co-localization of PtpA, which was shown to inhibit phago-lysosome fusion and neutralization of phagosome acidification in macrophage [14,23,28]. The potential role of PtpA and other virulence factors in DC infection and survival remain uncertain.

Infection of moDCs via MAP might be driven by DC-SIGN, which was shown to be the potential receptor for Mtb [40], although we have not investigated the responsible receptor for MAP uptake in this current study. One would expect that infection of DCs via MAP would likely result in the upregulation of chemokine receptors, allowing the migration of MAP-infected DCs to home to secondary lymphoid organs, to further spread the infection. However, a recent study demonstrated that Mtb infection of primary human DCs altered DC adhesion molecules and subsequent migration, via a downregulation of CD18, CD11a, and CD11b [41], which was in agreement with our data that demonstrated a significant decrease in CD11b, but not CD11c. This suggests that MAP prioritizes replication initially by delaying the initial adaptive immune responses. We showed that MAP infection and proliferation led to a delay in moDC maturation, as shown by an increase of anti-inflammatory cytokine IL-10 and CD103—a tolerogenic marker—at the time of infection, which we speculated might be caused by MAP’s ability to inhibit phago-lysosome fusion. As infection persisted, we observed an increase in the cytokine TNFα; a decrease in the cytokine IL-6; an increase in T-cell priming co-receptors CD80/86, and MHCII; and a decrease in CD103 and IL-10. Taken together, these data suggest that while moDCs recognize inflammatory pathogen-associated molecular patterns (PAMPs) on MAP, the live microorganisms might inhibit the inflammatory response. This could be a virulence mechanism through which MAP could evade antigen presentation, while allowing for spread to lymph nodes and other tissues, upon infection of DCs. We were also able to measure the percentage of moDCs infected with MAP by performing a phagocytosis assay. Our data resembled prior studies that demonstrated a 20% macrophage infection rate with MAP [42,43,44], where the moDCs in our study phagocytosed up to 18%. However, a new study [44] that used an MOI of 5:1 and 20:1, demonstrated a 16.25% and 38.78% infection rate, respectively. Our infection rates were comparable to those of a 5:1 MOI, as seen in the macrophage, which suggest that moDCs might have an increased ability to phagocytose bacteria compared to macrophage. As the infection rate was modest, we were therefore likely underestimating the magnitude of the moDC response in individually infected cells by looking at population MFIs of the surface markers. Nonetheless, we were able to measure robust inflammatory responses in the overall MAP-infected population.

One limitation to our study was the option to use moDCs over whole blood or gut tissue isolated DCs, to measure infection, proliferation, and phagocytosis. However, moDCs are widely used and provide a very useful model in the functional studies of immunology and infection [27,45,46,47], whereas human DCs isolated from whole blood are too rare to provide enough cells to test our hypothesis, and it is extremely difficult to obtain substantial numbers of tissue-resident DCs from intestinal biopsies. Another limitation is the inability to separate infected from uninfected moDCs in our assays, meaning that some of the cytokine and surface marker changes we observed could be due to the paracrine effects of the factors released from the MAP-infected cells on the uninfected cells. Nonetheless, our findings suggest that live MAP might have direct inhibitory effects on moDC inflammatory responses, which needs to be verified in future, larger studies.

## 5. Conclusions

We demonstrated that MAP infects and replicates inside of moDCs. This leads to an initial anti-inflammatory phenotype, which allows MAP to replicate, and then subsequently leads to a pro-inflammatory phenotype of CD80/86 expression and cytokine production, which would increase the moDCs’ ability to activate T-cells and present potential MAP antigens for subsequent MAP specific T-cell clonal expansion. The net result of this would be initial infection, persistence, and systemic dissemination of MAP, along with the later development of potentially damaging T-cell responses that could drive inflammatory injury in the gut of patients with CD who carry MAP.

## Supplementary Materials

Supplementary materials can be found at https://www.mdpi.com/2076-2607/8/7/994/s1. Figure S1. Flow cytometry gating strategy. Human moDCs at *T* = 0, 24, or 48 h after MAP infection were stained, as described in the Methods, and gated sequentially on forward/side scatter, single cells (SSC-A/SSC-H), viability, and finally CD11b^+^ and CD11c^+^ cells, prior to calculation of MFIs for CD80, CD86, MHCII, CD103, CD11b, and CD11c. Figure S2. Cell viability post MAP infection. The percentage of live moDCs untreated or post MAP/KMAP infection was calculated following forward/side scatter, CD11b^+^ and CD11c^+^, and gated on SSC-A and BV510 (viability dye). * *p* < 0.05 as calculated using a two-way ANOVA with multiple comparisons, shown is the average ± SEM of *n* = 4 DC blood donors in triplicates. Figure S3. Flow cytometry MFI shifts. Human MoDCs at *T* = 0, 24, or 48 h after the MAP infection were stained, as described in Methods, and gated sequentially on forward/side scatter, single cells (SSC-A/SSC-H), viability, and finally CD11b^+^ and CD11c^+^ cells. MFI shifts of a representative experiment at *T* = 24 are shown.
