# Supplementary material for: Mycobacterium avium Subspecies paratuberculosis Infects and Replicates within Human Monocyte-Derived Dendritic Cells
Source: Microorganisms. 2020 Jul 3;8(7):994. doi: 10.3390/microorganisms8070994 (PMC7409171; doi:10.3390/microorganisms8070994)
Supplement: Supplementary file 1 [file microorganisms-08-00994-s001.pdf]

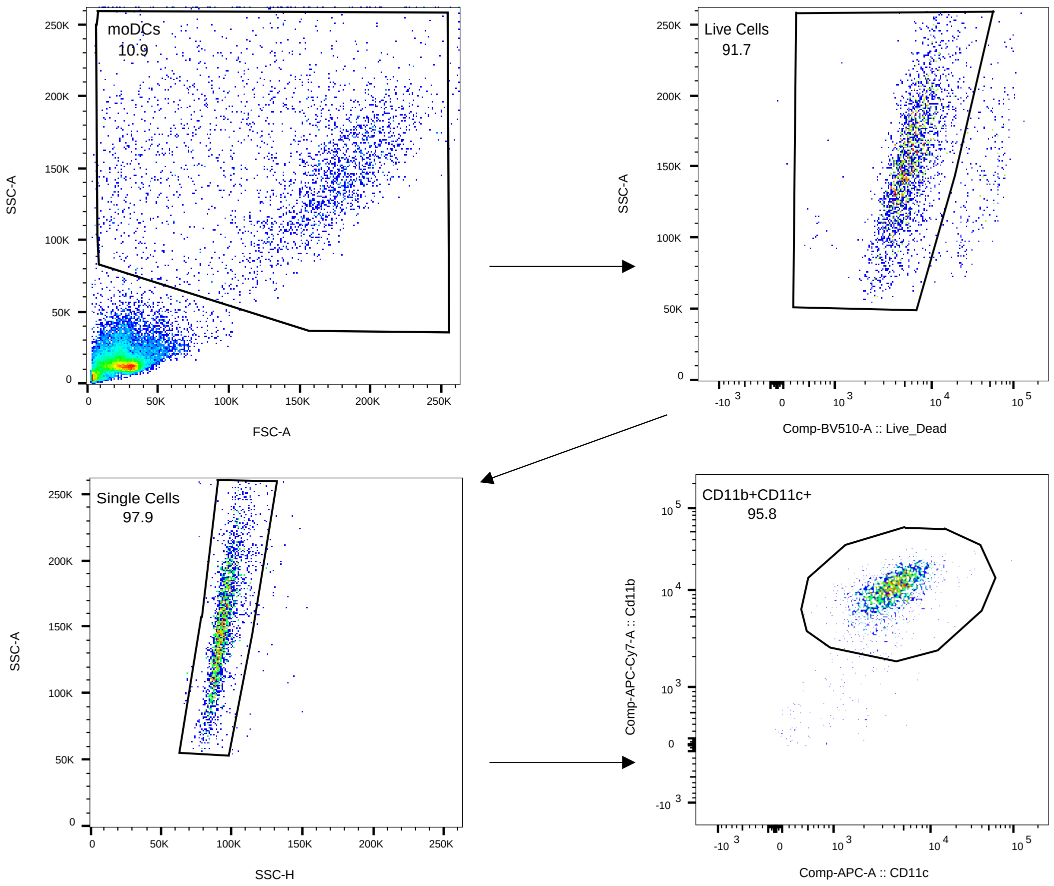

Figure S1. Flow cytometry gating strategy. Human moDCs at $T = 0$, 24, or 48 h after MAP infection were stained, as described in the Methods, and gated sequentially on forward/side scatter, single cells (SSC-A/SSC-H), viability, and finally CD11b$^+$ and CD11c$^+$ cells, prior to calculation of MFIs for CD80, CD86, MHCII, CD103, CD11b, and CD11c.

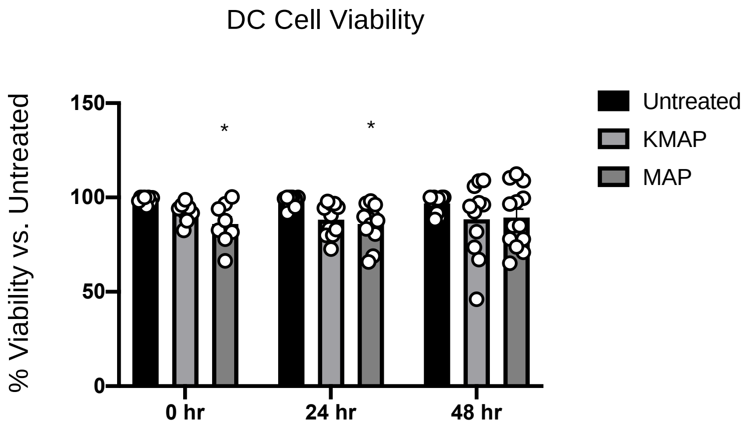

Figure S2. Cell viability post MAP infection. The percentage of live moDCs untreated or post MAP/KMAP infection was calculated following forward/side scatter, CD11b$^+$ and CD11c$^+$, and gated on SSC-A and BV510 (viability dye). * $p < 0.05$ as calculated using a two-way ANOVA with multiple comparisons, shown is the average ± SEM of $n = 4$ DC blood donors in triplicates.

Fig S3

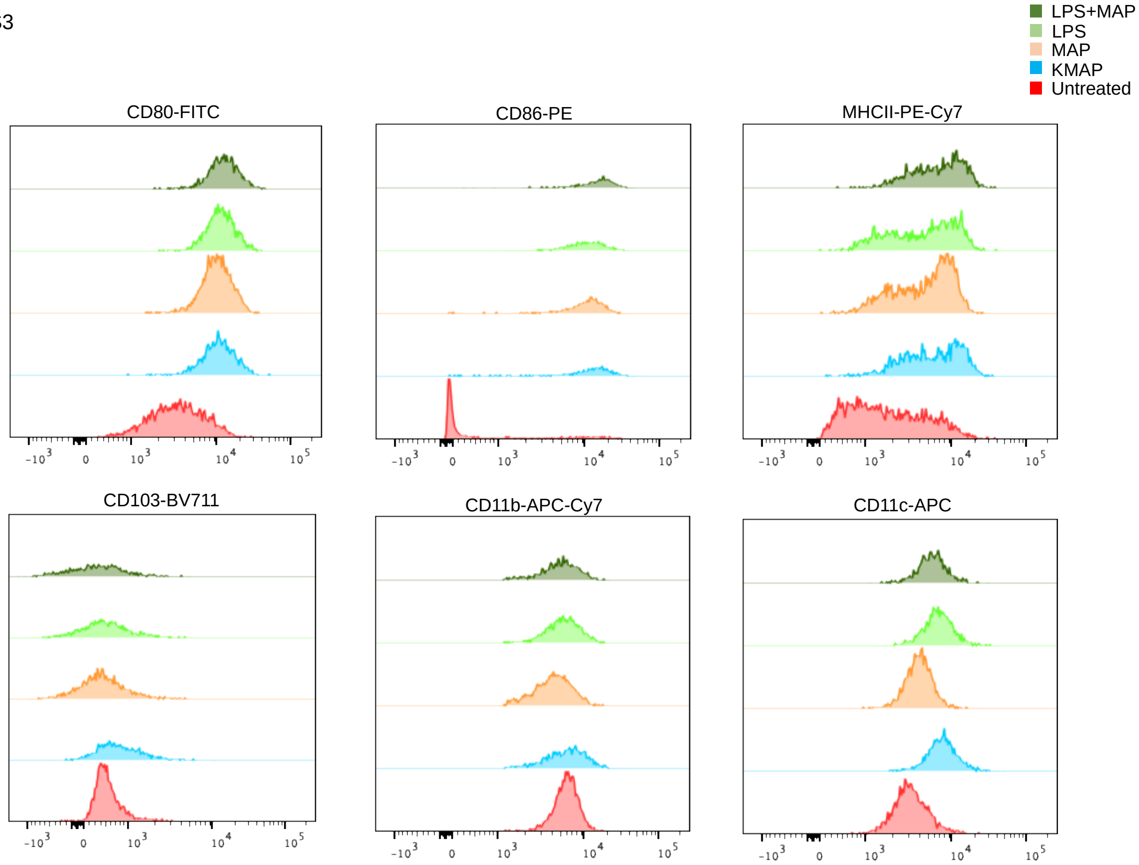

Figure S3. Flow cytometry MFI shifts. Human MoDCs at $T = 0$, 24, or 48 h after the MAP infection were stained, as described in Methods, and gated sequentially on forward/side scatter, single cells (SSC-A/SSC-H), viability, and finally $CD11b^{+}$ and $CD11c^{+}$ cells. MFI shifts of a representative experiment at $T = 24$ are shown.
